# Nuclear accumulation and activation of p53 in embryonic stem cells after DNA damage

**DOI:** 10.1186/1471-2121-10-46

**Published:** 2009-06-17

**Authors:** Valeriya Solozobova, Alexandra Rolletschek, Christine Blattner

**Affiliations:** 1 Institute of Toxicology and Genetics, Forschungszentrum Karlsruhe, PO-Box 3640, 76021 Karlsruhe, Germany; 2 Institute for Biological Interfaces, Forschungszentrum Karlsruhe, PO-Box 3640, 76021 Karlsruhe, Germany

## Abstract

**Background:**

P53 is a key tumor suppressor protein. In response to DNA damage, p53 accumulates to high levels in differentiated cells and activates target genes that initiate cell cycle arrest and apoptosis. Since stem cells provide the proliferative cell pool within organisms, an efficient DNA damage response is crucial.

**Results:**

In proliferating embryonic stem cells, p53 is localized predominantly in the cytoplasm. DNA damage-induced nuclear accumulation of p53 in embryonic stem cells activates transcription of the target genes *mdm2*, *p21*, *puma *and *noxa*. We observed bi-phasic kinetics for nuclear accumulation of p53 after ionizing radiation. During the first wave of nuclear accumulation, p53 levels were increased and the p53 target genes *mdm2*, *p21 *and *puma *were transcribed. Transcription of *noxa *correlated with the second wave of nuclear accumulation. Transcriptional activation of p53 target genes resulted in an increased amount of proteins with the exception of p21. While p21 transcripts were efficiently translated in 3T3 cells, we failed to see an increase in p21 protein levels after IR in embryonal stem cells.

**Conclusion:**

In embryonic stem cells where (anti-proliferative) p53 activity is not necessary, or even unfavorable, p53 is retained in the cytoplasm and prevented from activating its target genes. However, if its activity is beneficial or required, p53 is allowed to accumulate in the nucleus and activates its target genes, even in embryonic stem cells.

## Background

Cells are continuously subjected to DNA lesions arising both from environmental conditions and from the intrinsic metabolism of a cell. Such lesions can lead to mutations and large-scale genome alterations that may be deleterious for cellular function. To maintain genomic stability cell cycle checkpoints exist that can detect errors during DNA replication. If errors are encountered, cell division is paused and repair mechanisms and/or cell death ensues. The p53 tumor suppressor protein plays an important role in this process [1]. By being part of a signal transduction process, p53 relays information leading to cellular responses such as cell cycle arrest and apoptosis, resulting from DNA lesions. P53 activity is regulated mainly at the protein level. In response to DNA lesions, p53 is rescued from targeted degradation, which leads to a strong increase in the amount of the otherwise short-lived tumor suppressor protein, and the protein is intensively modified [2,3]. Cells deficient in p53 fail to undergo apoptosis or cell cycle arrest in response to DNA damage [4,5] which increases the rates of tumorigenicity and genomic instability in these animals [6-8].

Pluripotent, undifferentiated embryonic stem (ES) cells retain the potential to produce any cell type in the body and contribute to all adult cell lineages. While mutations in a somatic cell are limited to a particular lineage and do not affect progeny, ES cell mutations potentially compromise multiple lineages and affect the well-being of subsequent generations. As such, ES cells should have a highly sensitive and finely tuned response to DNA damage. In fact, previous reports described a reduction of mutation frequency by about two orders of magnitude in ES cells and a strongly enhanced sensitivity to ionizing radiation (IR) and other DNA damaging agents [9-12]. Because of the prominent role that p53 has in the DNA damage response of differentiated cells, it is most likely that p53 has a similar function in stem cells. Indeed, it has been reported that p53 deficiency increases the teratogenicity of mice after administration of benzo[a]pyrene or after irradiation [13,14].

Despite previous research, it is still unclear whether p53 is activated or not in stem cells in response to DNA damage. While Yang Xu and co-workers found that p53 levels are increased in response to DNA damage in ES cells and *mdm2 *and *noxa *are expressed after irradiation of ES cells with UV-light or treatment with doxorubicin, Mirit Aladjem observed that p53 failed to activate a stress response in ES cells after treatment with PALA, IR or adriamycin despite the accumulation of the tumor suppressor protein in the nucleus of DNA-damaged ES cells, [15,16].

To clarify about the role of p53 in ES cells, we investigated p53 localization and activity in resting ES cells and in ES cells subjected to DNA damage in more detail. Similar to Mirit Aladjem and co-workers [16], we found the majority of p53 localized in the cytoplasm of proliferating ES cells. However, after irradiation, p53 accumulated in the nucleus. Nuclear accumulation of p53 in irradiated stem cells correlated with the activation of *mdm2*, *p21*, *noxa *and *puma *and the suppression of *nanog*.

## Results

### P53 is localized to the cytoplasm of mouse ES cells

**Figure 1:**
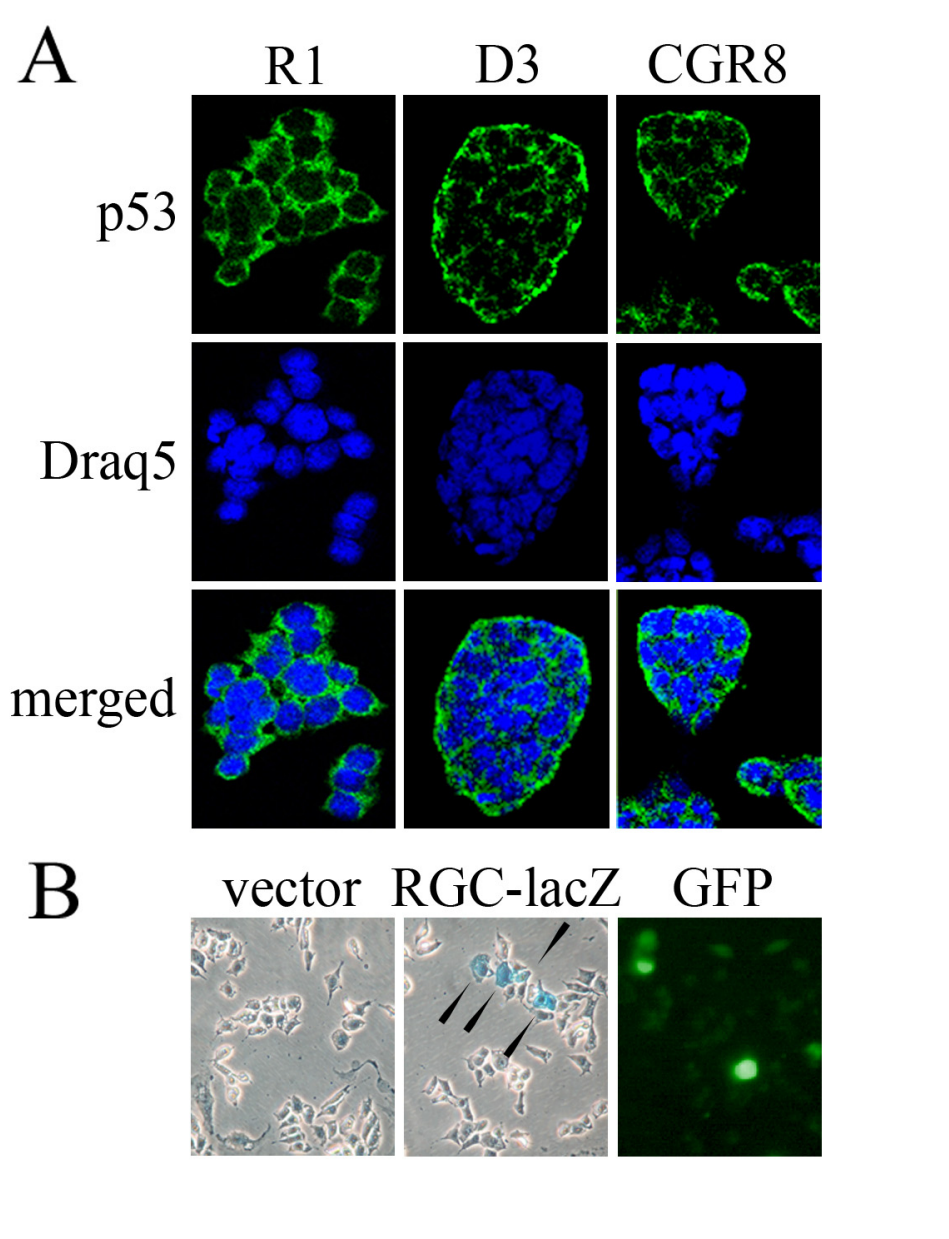
**Cytoplasmic localisation of p53 in proliferating ES cells**. R1, D3 and CGR8 mouse embryonal stem cells were plated on cover slips (R1 and D3 in the presence of feeders, CGR8 in the absence of feeders). Two days after plating, cells were fixed in acetone/methanol, permeabilized with Triton-X-100 and incubated with the anti-p53 antibody Pab 421. After incubation, cover slips were washed and incubated with an antibody directed against mouse IgG, coupled to Alexa-Fluor-488 (green). To visualize the nuclei, cover slips were incubated with Draq5 (blue). **(B) **R1 ES cells were transfected with RGC-lacZ or with a vector control together with GFP to visualize transfection efficiency. Two days after transfection, cells were fixed with paraformaldehyde and incubated with X-gal in reaction buffer to monitor β-galactosidase expression. Arrows point to lacZ expressing R1 cells.

In an attempt to resolve the caveat whether p53 is active or not in stem cells, we first determined the localization of the p53 protein in mouse ES cells. We investigated three different mouse ES cell lines, R1, D3 and CGR8 and determined p53 localization by immuno-fluorescence staining. R1 and D3 cells were cultured on feeder cells while CGR8 stem cells do not require feeders for maintaining an undifferentiated phenotype. In consistency with a previous report [16], we found the majority of p53 localized in the cytoplasm (Figure 1A, Additional file 1). These results however do not exclude the possibility that a small fraction of p53 exists in the nucleus.

Since some p53 isoforms have been shown to be cytoplasmic [17], we wondered whether p53 might show cytoplasmic localization in murine ES cells because of the expression of particular cytoplasmic isoform of p53. However, both RT-PCR and Western Blotting with a polyclonal anti-murine p53 antiserum, detected a single strong band for p53. This band migrated app. with the 53 kDa marker in SDS gels and with the 1200 bp marker in agarose gels (Additional file 2). Only upon longer exposures, smaller bands became visible. Whether these smaller bands represent authentic splice products of p53 or whether they only show cross-reactivity with other factors remains to be elucidated.

As a transcription factor, p53 usually needs to be nuclear to be active. Because of its cytoplasmic expression, we wondered whether p53 would be able to activate transcription of its target genes in ES cells. To test this possibility we transfected R1 ES cells with a cDNA encoding the lacZ gene under the control of the p53-responsive RGC promoter [18] and determined expression of β-galactosidase. For monitoring transfection efficiency, we co-transfected a cDNA encoding the green fluorescent protein (GFP). Only a few cells showed β-galactosidase expression (Figure 1B). Likewise, only a few cells showed expression of GFP which indicates that a minority of stem cells had been transfected. Since staining for GFP and X-gal corresponded in numbers, it is most likely that all transfected ES cells express β-galactosidase under the control of p53.

### P53 becomes activated in ES cells after DNA damage

**Figure 2:**
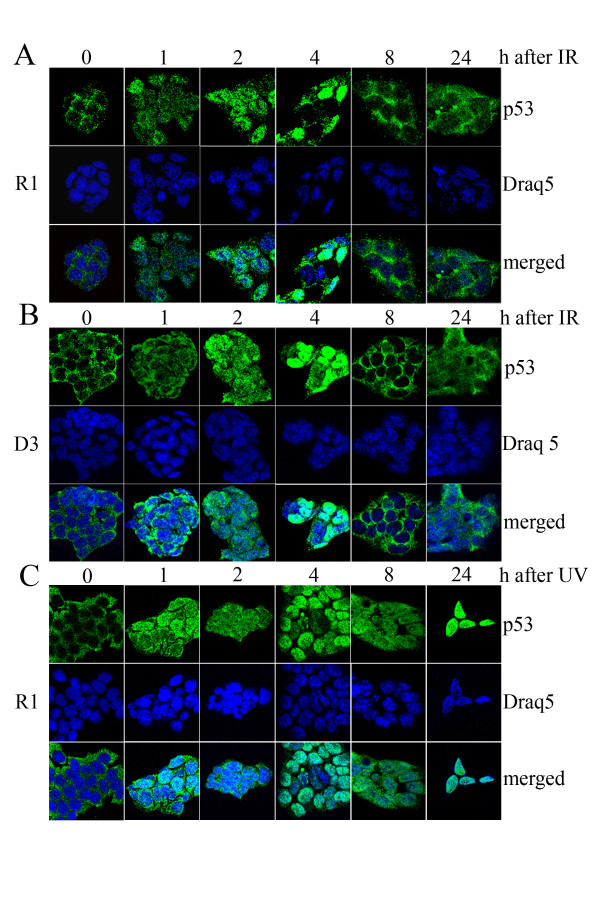
**p53 accumulates in the nucleus of irradiated ES cells**. R1 **(A, C) **and D3 **(B) **stem cells, grown on feeders, were irradiated two days after plating with 7.5 Gray IR **(A, B) **or with 30 J/m2 UVC light **(C) **and harvested at the indicated times. Cells were fixed in acetone/methanol, permeabilized with Triton-X-100 and incubated with the anti-p53 antibody Pab 421. After incubation, cover slips were washed and incubated with an antibody directed against mouse IgG, coupled to Alexa-Fluor-488 (green). To visualize the nuclei, cover slips were incubated with Draq5 (blue).

One particularly important function of p53 is DNA damage signaling [4,5]. Here, to suppress tumorigenesis, p53 halts the cell cycle and induces apoptosis in primary cells and in tumor cell lines. Since stem cells provide the pool of proliferative pluri/toti/omni-potent cells within organisms, they are more likely to propagate DNA lesions and mutations to daughter cells compared to differentiated cells. We therefore speculated that p53 has an important role in the DNA damage response of stem cells. Since p53 is primarily a transcription factor, nuclear localization of p53 should be essential for its transcriptional activity. This activity is, however, not consistent with the cytoplasmic localization of the tumor suppressor protein in ES cells. One possibility could be that despite its cytoplasmic localization in non-stressed ES cells, p53 accumulates in the nucleus of ES cells after DNA damage. We tested this possibility after irradiation with both IR and UV light. As we show already in figure 1, in unstressed ES cells p53 was localized mainly to the cytoplasm. However, at one hour after irradiation, p53 accumulated in the nucleus of irradiated ES cells (Figure 2A, B). At four hours after irradiation, p53 was still present in the nucleus of some cells, while in others it had mostly disappeared. At eight hours after irradiation p53 had essentially disappeared from the nucleus of all cells. Surprisingly, at 24 hours after IR, p53 was again localized in the nucleus of most cells. Nevertheless, a minority of cells still showed cytoplasmic localization of p53 (Figure 2A, B). The same biphasic accumulation of p53 in the nucleus of irradiated cells was also observed in the CGR8 stem cell line (data not shown).

After UV-irradiation, p53 also accumulated in the nucleus of ES cells. In contrast to IR-irradiated cells, p53 remained in the nucleus of UV-irradiated cells. At 24 hours, most of the ES cells had died while the few remaining ones showed a very intensive nuclear staining for p53 (Figure 2C).

**Figure 3:**
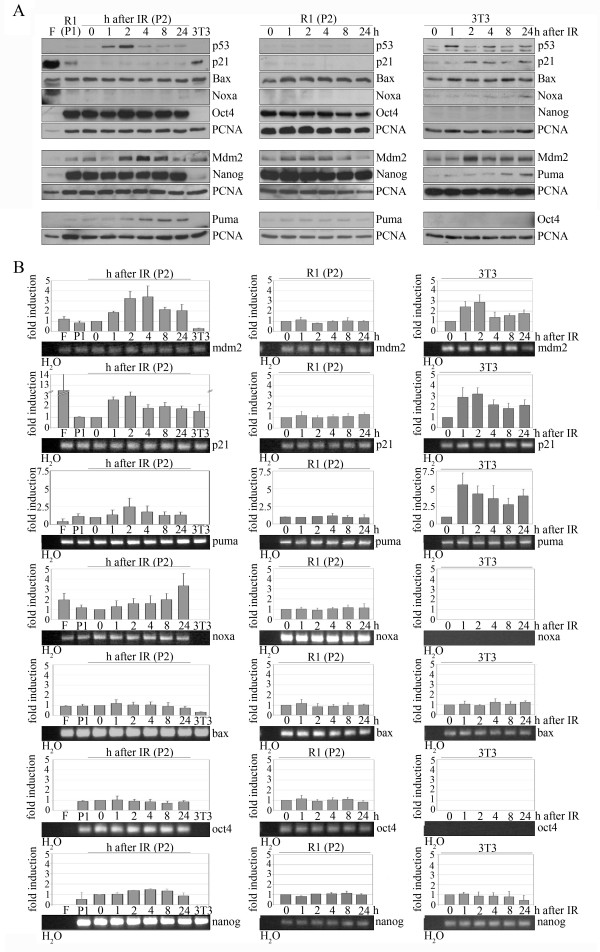
**p53 is activated in ES cells after IR**. R1 stem cells passaged once (P1) or twice (P2) without feeders and 3T3 mouse fibroblasts were irradiated with 7.5 Gray or left unirradiated. Cells were harvested at the indicated times and divided into two aliquots. One of the aliquots was used to determine the amount of protein (A) and the second aliquot (B) to monitor the abundance of corresponding RNAs. **(A) **Western Blots showing p53, p21, Bax, Noxa, Mdm2, Puma, Oct4, Nanog and PCNA (proliferating cell nuclear antigen) for loading control. **(B) **Real time PCR for *mdm2*, *p21*, *puma*, *noxa*, *bax*, *oct4 *and *nanog *RNA. Mean values and standard deviations were calculated from the obtained 2^dCT numbers of qRT-PCR signals of 3–6 independent experiments and blotted. Values of non-irradiated P2 cells at time point "0" and non-irradiated 3T3 cells, respectively, were set to 1. The quality of the qRT-PCR products was controlled by agarose gel electrophoresis. A representative picture of the gel is placed below the graph.

After having observed that p53 accumulates in the nucleus of irradiated ES cells, we used qRT-PCR and Western Blotting to test whether p53 activated transcription of its target genes. To prevent contamination by feeder cells, ES cells were sub-cultured once (P1) or twice (P2) without feeder cells. Since ES cells were sub-cultured every day, P2 cells had been cultured without feeders for only two days. To exclude the possibility of premature differentiation in the absence of feeder cells, we monitored expression of *nanog *and *oct 4 *at the RNA and protein level (Figure 3A, B) and of alkaline phosphatase (data not shown). Although P2 cells were hardly contaminated with feeder cells, we additionally determined expression of p53 target genes in feeder cells. We further included 3T3 mouse fibroblasts in the analysis in order to compare the regulation of p53 target genes in ES cells with that of differentiated cells. To exclude artifacts due to culture conditions, we also included ES (P2) cells that had not been irradiated but were plated and harvested at the same time as irradiated cells. As we show in figure 3A, the accumulation of p53 in the nucleus of irradiated ES cells (compare Figure 2A) was accompanied by a strong increase in the amount of p53 protein. Shortly after the accumulation of p53 protein levels, Mdm2 and Puma proteins levels also increased. Consistent with a previous report [16], the amount of p21 protein was very low in stem cells and was not increased after irradiation. Likewise, we failed to detect an increase in the amount of Bax protein. We also observed an increase in the amount of Noxa protein, but only at 24 hours and induction was very low. Consistent with the study by Lin and co-workers [15], we found that Nanog protein was slightly reduced after DNA damage, but recovered to basal levels eight hours after irradiation (Figure 3A). In non-irradiated R1 cells, the levels of the investigated proteins remained unchanged throughout the experiment (Figure 3A). In comparison to ES cells, 3T3 cells showed slightly different kinetics of p53 induction after IR. Here, we observed the strongest induction at one hour after irradiation. Consistent with a more important role of p53 for the maintenance of genomic integrity in stem cells than in differentiated cells, the overall induction of p53 was much higher in ES cells than in 3T3 cells. Like in ES cells, the increase in the amount of p53 in 3T3 cells was followed by an increase in the amount of the Mdm2 and the p21 protein. The amount of the p21 protein was also very high in feeder cells which have been irradiated prior to the plating of the cells, suggesting that p21 levels may remain high for several days after irradiation. As expected, we failed to detect Nanog and Oct-4 proteins in 3T3 cells (Figure 3A). The determination of the amount of proteins was complemented by qRT-PCR. To allow a direct comparison of the expression of p53 target genes at the RNA and protein level, we performed qRT-PCR on a second aliquot of the same lot of cells that had been used for Western Blot analysis. The analysis of the PCR products by agarose gel electrophoresis revealed single bands in all cases (Figure 3B). It should, though, be noted that the analysis of the PCR products by agarose gel electrophoresis was not quantitative. The analysis of gene expression by qRT-PCR essentially mirrored results from Western blotting, with two exceptions: While the p21 protein was hardly detectable in stem cells and its amount was not increased after IR, we observed *p21 *RNA in stem cells at a level that was almost comparable with the amount of *p21 *RNA in 3T3 cells. Moreover, the amount of *p21 *RNA was further increased in response to IR in ES cells. Another difference between protein and RNA analysis was observed for Puma. While Western blotting displayed a similar increase in the amount of Puma in 3T3 and ES cells, *puma *RNA was induced about 2-fold stronger in 3T3 cells than in ES cells. Like the protein, *mdm2 *RNA accumulated transiently in both ES and 3T3 cells. However, maximum *mdm2 *induction in 3T3 cells occurred earlier (Figure 3B), consistent with the earlier increase in p53 protein abundance (Figure 3A). Consistent with the second phase of nuclear accumulation of p53, we observed induction of the p53 target gene *noxa *at twenty-four hours after irradiation in ES cells while we failed to detect expression of noxa in 3T3 cells, despite its expression in feeder cells. We also failed to detect an increase in the expression of *bax *RNA both in ES cells and in 3T3 cells. Both stem cell markers, *oct 4 *and *nanog *were highly expressed in ES cells but clearly reduced (*nanog*) or absent (*oct 4*) in differentiated cells. After irradiation, the amount of *nanog *RNA was slightly reduced in ES cells but not in 3T3 cells (Figure. 3B).

### Influence of p53 on proliferation and survival of ES cells after ionising radiation

**Figure 4:**
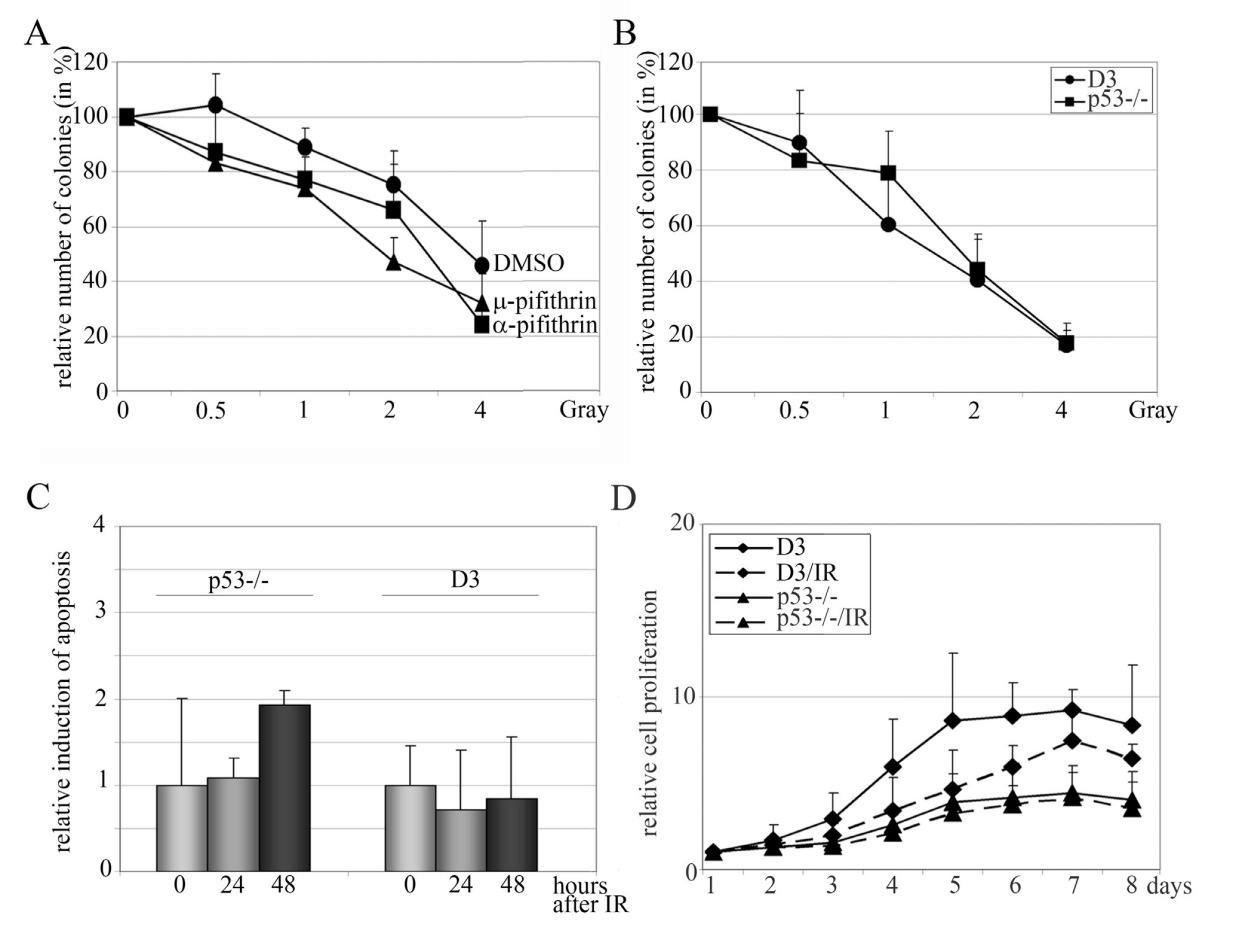
**Proliferation and apoptosis assays of ES cells in the presence and absence of active p53**. **(A) **R1 cells were treated with 10 μM α-pifithrin, μ-pifithrin or carrier 24 hours after plating. Two hours after drug addition, cells were irradiated and after additional two hours, the medium was replaced with normal growth medium. After two weeks, colonies were counted. The graph shows mean values and standard deviations of three to six independent experiments. The number of colonies from non-irradiated cells was set to 100%. **(B) **D3 or p53^-/- ^ES cells were irradiated with IR 24 hours after plating. After two weeks, colonies were counted. The graph shows mean values and standard deviations of three independent experiments. The number of colonies from non-irradiated cells was set to 100%. **(C) **P53-/- cells and D3 cells passaged once without feeders (P1 cells) were irradiated with 2 Gy and analysed for Annexin V staining after 24 and 48 hours. The graph shows the relative number and standard deviation of Annexin-V positive cells of three independent experiments. **(D) **D3 or p53-/- ES cells were irradiated with 2 Gray of IR and analysed at the indicated times by MTT assay. Mean values and standard deviations were calculated and plotted. The numbers for the two cell lines and conditions at day one were set to 1.

After showing that p53 accumulates in the nucleus and activates transcription of its target genes, we wondered whether the presence of p53 might be required for a normal DNA damage response in ES cells. To address this question, we first compared the colony forming ability of R1 ES cells with that of R1 ES cells where p53 activity was inhibited by treatment with α- or μ-pifithrin [19,20]. As we show in figure 4A, irradiation reduced the colony forming ability of R1 ES cells in a dose dependent manner. Treatment with pifithrin further sensitized the ES cells to IR, suggesting a protective effect of p53. To further investigate this protective effect, we repeated the colony forming assay with p53-negative ES cells (p53-/-) and the corresponding parental cell line (D3). Both cell lines showed a dose dependent decrease in their colony forming ability after IR. The sensitivity of D3 cells and the p53-negative derivatives to irradiation, though, appeared to be significantly stronger than that of R1 cells and the size of the individual colonies varied significantly after irradiation in both D3 cells and the p53-negative derivative. A protective effect of p53 was, however, not discernable (Figure 4B).

In apoptotic cells, the membrane-phospholipid phosphatidylserine is translocated from the inner to the outer surface of the plasma membrane, thereby exposing phosphatidylserine to the external cellular environment. The phospholipid-binding protein Annexin-V, which has a high affinity for the phospholipid binds to those cells that expose phosphatidylserine. This association of Annexin-V with exposed phosphatidylserine is a sensitive and widely accepted method for the determination of apoptosis. We applied this method to determine the influence of p53 on the initiation of apoptosis of irradiated ES cells. To prevent contamination, we performed this assay with ES cells that have been passaged once without feeder cells (P1 cells). Under these conditions, we observed no signs of apoptosis with p53-positive D3 cells while p53-negative cells showed a slight increase in the number of apoptotic cells at 48 hours after irradiation (Figure 4C). Nevertheless, the increase was still within the error bars of non-irradiated cells and unexpectedly low. We also performed this analysis with cells that had been passaged twice without feeders (P2 cells). Under these conditions about twice as many ES cells were Annexin-V-positive, even without irradiation (data not shown). This number of Annexin-V-positive ES cells was slightly increased after irradiation. Nevertheless, the increase in Annexin-V positive cells after irradiation never exceeded a factor of 3.5 (Additional file 3).

A widely used assay for the determination of relative cell proliferation and cell viability is the colorimetric MTT-assay, which determines the reduction of yellow (3-(4.5-Dimethylthiazol-2-yl)-2.5-diphenyltetrazolium bromide to purple formazan by metabolic enzymes. We employed this assay to determine relative cell proliferation and cell viability of irradiated p53-positive and the corresponding p53-negative ES cells. Surprisingly, p53-negative ES cells derived from the D3 ES cell line proliferated slower and reached a plateau at a lower cell density in comparison with their parental counterpart. Irradiation had only a minor impact on the proliferation of these p53-negative ES cells and affected cell number only within the first 48 hours after irradiation. Thereafter, irradiated and non-irradiated p53-negative ES cells had a similar proliferation rate. Proliferation of the corresponding p53-positive ES cell line was affected much more by radiation. Cell proliferation of the D3 ES cell line was more strongly reduced after irradiation and the proliferation rate was decreased over several days (Figure 4D).

## Discussion

Consistent with a previous report [16], we found p53 localized in the cytoplasm of ES cells. However, while Mirit Aladjam and co-workers reported that p53 is inactive in stem cells [16], we found that p53 activates transcription of a reporter gene in resting ES cells and of endogenous target genes in response to DNA damage. The activity of p53 in resting stem cells shows that the p53 protein is also in ES cells in a latent state and can be activated when its activity is required. This result is consistent with a study from Sabapathy et al., who observed binding of p53 from ES cells to oligonucleotides corresponding to the p53 consensus binding sequence [21]. After DNA damage, p53 did not activate all, but at least some of its target genes in ES cells. Transcription of these genes was facilitated by the presence of active p53 in the nucleus of irradiated cells. After IR, p53 accumulated in two waves in the nucleus of ES cells. The first nuclear accumulation occurred at one to two hours after irradiation and correlated with an increase in the amount of the p53 protein. During the second wave of nuclear accumulation of p53 at twenty-four hours after irradiation, we did not see an increase in p53 abundance suggesting that for the second wave of nuclear accumulation p53 was translocated from the cytoplasm into the nucleus. Whether nuclear translocation also contributed to the first wave of nuclear accumulation of p53 is still unclear. Both waves of nuclear accumulation of p53 corresponded to transcriptional activation of target genes. The first wave of nuclear accumulation of p53 correlated with transcriptional activation of *mdm2*, *p21 *and *puma *[22-24], while *noxa *[25] was transcribed during the second wave of nuclear accumulation of p53. Why some of the p53 target genes are transcribed only during the first wave of nuclear accumulation and why at least one other gene is transcribed only during the second wave of nuclear accumulation is presently unclear. It may be that distinct post-translational modifications of p53 are induced with different kinetics after irradiation and are required for the activation of different p53 target genes [26].

Most surprisingly, p21 was actively transcribed in ES cells and the amount of its RNA was increased further in response to IR, while the protein was hardly detectable before and after irradiation. This result indicates that production of p21 protein is strongly regulated at a post-transcriptional level in ES cells. Eventually, ES cells with damaged DNA require efficient elimination from the population. Since G1 arrest triggered by p21 can prevent cells from S-phase entry and thus from the cellular death program that is executed in the S-phase of cells with damaged DNA, e.g. by the activity of proteins such as Killin, the presence of p21 might counteract this elimination [27,28]. However, consistent with the results of Corbet et al., we did not see a strong induction of apoptosis in ES cells after ionizing radiation, as revealed by Annexin-V staining [29] which argues against this possibility.

While we observed activation of the transcription of *mdm2*, *puma*, *noxa *and *p21*, we failed to detect an activation of *bax *transcription [30] after IR. Probably, activation of bax after DNA damage is restricted to specific cell types that were not included in this study. Such a selectivity of gene expression in different cell lines is known for several years. For instance, it had been shown that γ-irradiation leads to a prolonged cell cycle arrest in fibroblasts whereas the same challenge leads to apoptosis in thymocytes and intestinal epithelial cells [31-34].

Overall, the impact of p53 on the response of murine ES cells to ionizing radiation was surprisingly low. The comparison of p53-positive and p53-negative stem cells of the same background showed basically identical colony forming abilities after DNA damage. Only when we treated the R1 ES cell line with chemical inhibitors of p53, we observed a slightly decreased survival in the presence of the drug. Since this result could not be reproduced with p53-positive and p53-negative ES cell lines, the increased sensitivity of R1 ES cells in the presence of μ- or α-pifithrin is most likely due to off-target effects. It should though be noted that Corbet et al., although they also found only very little induction of apoptosis after irradiation of ES cells with IR, reported a more pronounced reduction in the colony forming ability of p53-positive ES cells after IR than of p53-negative cells [29]. The reason for the discrepancy of our result and the result from Corbet is unclear. However, since the ES cell lines that were used by Corbet et al., and the ones that we used are not identical, it is likely that the different results are due to a variation in intrinsic cellular paramaters. Only when we applied the MTT assay, we observed a slight difference between p53-positive and p53-negative ES cells after irradiation. However, this result should be taken with care. First of all, the MTT-assay is a metabolic assay and conditions that affect cell metabolism can impact on the result. Secondly, p53-negative cells showed a significantly reduced proliferation (or metabolic) behavior compared to the p53-positive parental cell line. This different proliferation behavior could also affect the outcome of the assay.

While p53 is predominantly a nuclear protein in differentiated cells, in ES cells p53 is mostly cytoplasmic. The rational for this aberrant localization of p53 in ES cells is still unclear. However, since p53 suppresses nanog expression as well as self-renewal of adult neural stem cells [15,35] it is possible that p53 needs to be separated from the nucleus of ES cells to maintain an undifferentiated phenotype. By sequestering p53 to the cytoplasm, p53 is unable to suppress nanog while, at the same time, sufficient latent p53 protein is present in ES cells to enter the nucleo-cytoplasmic barrier when required.

## Conclusion

Our study clearly shows that p53 can be activated in stem cells despite its cytoplasmic localisation. However, results for p53 activation in stem cells depend on the selection of genes that are analyzed and whether the analysis is performed at the RNA or protein level.

Whether p53 activity is required for the DNA damage response of ES cells cannot be answered that easily. Overall, we found only a minor effect of p53 activity on proliferation and survival of ES cells after ionizing irradiation. The analysis, however, also shows that in dependence on what method is used, the result can vary.

## Methods

### Cell lines and their treatments

R1 and D3 ES cells were cultured in GlutaMAX™-I medium (Invitrogen) supplemented with 15% foetal bovine serum, 0.1 mM β-mercaptoethanol, 40 μg/ml gentamycin and 1000 units/ml LIF in culture dishes that had been coated with 0.1% gelatine. Mouse embryonal fibroblasts that had been irradiated with 6.3 Gray served as feeder cells. p53^-/- ^ES cells were cultured in GlutaMAX™-I medium (Invitrogen) supplemented with 15% foetal bovine serum, 0.1 mM β-mercaptoethanol, 1000 units/ml LIF and 300 μg/ml G418 in culture dishes that had been coated with 0.1% gelatine. SNL cells irradiated with 6.3 Gray served as feeder cells. CGR8 cells were grown in Glasgow Minimum Essential Medium (Sigma) supplemented with 10% foetal bovine serum, 40 μg/ml gentamycin, 100 units/ml LIF, 0.05 mM β-mercaptoethanol, 2 mM L-glutamine and 1 mM non-essential aminoacids without feeder cells. Culture dishes were coated with 0.2% gelatine. All ES cell lines were sub-cultured each day. 3T3 cells were grown in Dulbecco's Modified Eagle Medium (Invitrogen) supplemented with 10% donor bovine serum (Invitrogen) and 1% penicillin/streptomycin (Invitrogen). All cells were cultured at 37°C and 6% CO_2 _in a humidified atmosphere.

Cells were irradiated with a ^60^cobalt γ-ray source at a dose rate of 1 Gray per minute in cell culture medium. For UV-irradiation, the culture medium was removed and saved. The cells were washed with PBS and irradiated with 30 J/m^2^. After irradiation, the original culture medium was added back to the cells.

Transfections were performed using the mouse ES cell Nucleofector Kit (Amaxa) according to the manufacturer's recommendations.

### Antibodies

The mouse monoclonal anti-p53 antibody Pab421 was purchased from Biomol, the rabbit polyclonal anti-p53 antibody CM5 was bought from Vector Laboratories, the mouse monoclonal anti-Mdm2 antibody 4B2 was obtained from Calbiochem and the rabbit polyclonal anti-Bax and anti-Puma antibodies were from Cell Signalling. Antibodies targeted against p21 (C-19), Noxa (K-16), Oct-4 (C-10) and PCNA (PC10) were purchased from Santa Cruz. The antibody directed against Nanog was obtained from Millipore. Alexa-488 goat anti-mouse antibody (Invitrogen) and HRP-coupled goat anti-mouse and goat anti-rabbit antibodies (Dako) were used as secondary antibodies.

### SDS-PAGE and Western blotting

SDS-PAGE and Western blotting were performed as described [36].

### Immunofluorescence staining

ES cells were grown on feeder cells that had been grown on cover slips. After washing twice with ice cold PBS, cells were fixed with ice-cold acetone/methanol (1:1) for 8 min on ice. Cover slips were washed 3 times with PBS and cells were permeabilized with 0.5% Triton-X-100 in PBS for 10 min. Cover slips were washed 3 times with PBS and incubated for 30 min in blocking buffer (1% bovine serum albumin; 1% goat serum in PBS). After blocking, cells were incubated overnight with an antibody directed against p53 (Pab421) that had been diluted 1:200 in blocking buffer. Cover slips were washed 3 times with PBS and incubated for 30 min at room temperature in the dark with an antibody directed against mouse IgG coupled to Alexa-Fluor-488 (Invitrogen) and Draq 5 (Biostatus Limited) both diluted 1:1000 in blocking buffer. Cover slips were washed 3 times and mounted with Hydromount on microscope slides. Cells were analysed using a Zeiss LSM510 confocal microscope and LSM LSell5 Image Examiner software.

### RT-PCR

Total RNA was prepared from cells using the RNeasy kit (Qiagen) according to the manufacturer's recommendation and treated with DNase I to remove residual genomic DNA. RNA was transcribed into cDNA using random primers and RevertAid^tm ^H MinusM-MuLV reverse transcriptase (Fermentas). Real-time PCR was performed in duplicates with a SYBR Green PCR mixture (Qiagen). The cDNA was denatured for 15 min at 95°C followed by 40 cycles of 95°C for 15 s and 50°C for 1 min using the 7000 ABI sequence detection system and gene specific primers. The signals were normalized to the signals for the housekeeping gene 34B4. Sequences of primers are available on request.

### Beta-Galactosidase staining

Cells were washed with PBS and fixed with 3.5% paraformaldehyde in PBS for 10 min on ice. Cells were washed 3 times with PBS, incubated with X-gal (0.25 mg/ml) and solubilised in reaction buffer (5 mM potassium ferricyanide, 5 mM potassium ferrocyanide, 2 mM magnesium chloride in PBS) for 16 h at 37°C. Cells were analysed with a Zeiss Axiovert microscope.

### MTT-assay

10^6 ^D3 cells or p53-/- cells were plated on 0.1% gelatine-coated 6 cm-plates. After attachment, cells were irradiated with 2 Gray or left unirradiated for control. Each day, MTT (*3*-[4,5-Dimethylthiazol-2-yl]-2,5-diphenyltetrazolium Bromide) was added to two plates at a final concentration of 2.5 mg/ml and incubated for 4 h. The reaction was stopped by removal of the medium and solubilization of the MTT-precipitate in 2 ml isopropanol. Absorbances were read at 595 nm.

### Colony assay

200 ES cells were plated in 3.5 cm dishes coated with 0.2% gelatine. Twenty-four hours after plating α-pifithrin and μ-pifithrin were added to a final concentration of 10 μM. Two hours after drug addition, cells were irradiated with 0.5 Gy, 1 Gy, 2 Gy and 4 Gy. After additional two hours, the culture medium was replaced with fresh medium without inhibitors and the cells were incubated for two weeks with a daily change of culture medium. The cells were washed with PBS, fixed with methanol, stained with 1% crystal violet in PBS and counted. For colony assays in the absence of an inhibitor, cells were irradiated at four hours after plating and incubated for two weeks with a daily change of culture medium.

### Apoptosis Assay by Annexin V staining

1 × 10^6 ^D3 or p53-/- ES cells were washed with ice-cold PBS and resuspended in 400 ml Ca-containing buffer (10 mM HEPES, pH 7.4, 140 mM NaCl, 5 mM CaCl_2_). 5 μl of annexin V-FITC (BD Pharmingen) and 1 μg propidium iodide were added for 10 min and the cells were immediately analyzed by a BD FACScan Flow Cytometer (Becton Dickinson).

## Authors' contributions

VS performed cell culture and carried out immunofluorescence staining, RT-PCR, β-galactosidase staining, colony assays, MTT-assays and the apoptosis assay. AR participated in the design of the study and supervised and revised the ES cell work. CB performed Western blotting, designed the study and drafted the manuscript. All authors read and approved the final manuscript.

## Supplementary Material

Additional file 1 **Cytoplasmic localization of p53 in ES cells**. R1, D3 and CGR8 mouse embryonal stem cells were plated on cover slips (R1 and D3 in the presence of feeders, CGR8 in the absence of feeders). Two days after plating, cells were fixed in acetone/methanol, permeabilized with Triton-X-100 and incubated with the anti-p53 antibody Pab 421 or only with blocking buffer, for control. After incubation, cover slips were washed and incubated with an antibody directed against mouse IgG, coupled to Alexa-Fluor-488 (green). To visualize the nuclei, cover slips were incubated with Draq5 (blue). Click here for file

Additional file 2 **The 53 kDa-form is the major form of p53 in mouse ES cells**. (A) R1, D3 and 3T3 cells, for control, were lysed and 50 μg of the proteins were separated by a 10% SDS-PAGE gel. The proteins were blotted onto an Immobilone P membrane and hybridised with the polyclonal anti-p53 antibody CM5. After washing and hybridisation with an anti-rabbit antibody coupled to HRP, the blot was developed by ECL. (B) Total RNA was prepared from D3, R1 and 3T3 cells, for control, and transcribed into cDNA. PCR was performed using gene specific primers for murine p53 and analysed by agarose gel electrophoresis. Click here for file

Additional file 3 **Annexin-V staining of irradiated p53-/- and p53+/+ ES cells**. P53-/- cells and D3 cells that had been passaged twice without feeders (P2 cells) have been irradiated with 2 Gy and harvested after 24 and 48 hours. Cells were resuspended in Ca-containing buffer in the presence of Annexin V-FITC and propidium iodide and analysed by a BD FACScan Flow Cytometer. The data shown in the graph represent the relative number and standard deviation of Annexin-V positive cells of three independent experiments. Click here for file
