# Supplementary figures and images for: Nuclear accumulation and activation of p53 in embryonic stem cells after DNA damage
Source: BMC Cell Biol. 2009 Jun 17;10:46. doi: 10.1186/1471-2121-10-46 (PMC2704172; doi:10.1186/1471-2121-10-46)

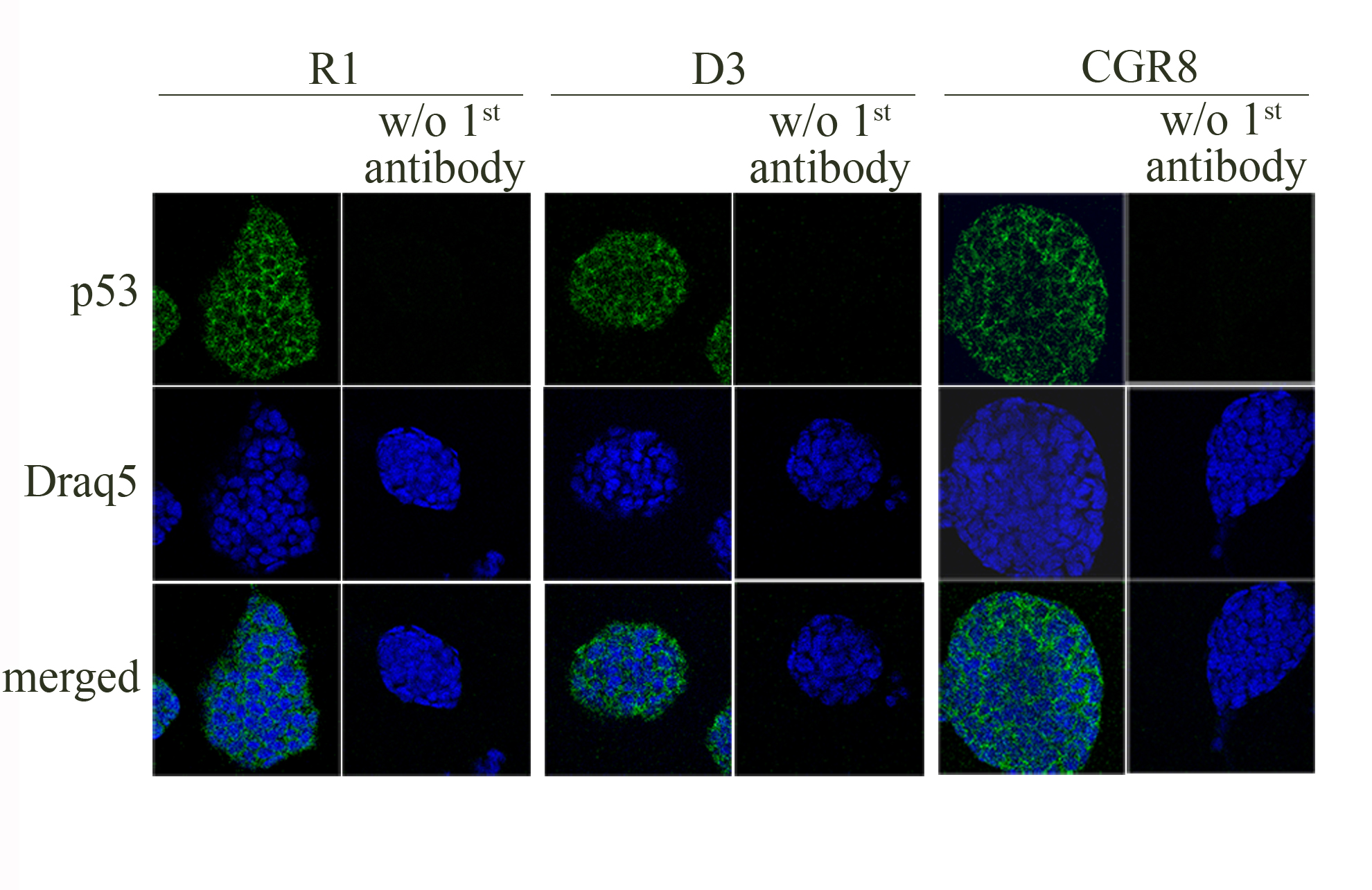

Supplement: Additional file 1 — Cytoplasmic localization of p53 in ES cells. R1, D3 and CGR8 mouse embryonal stem cells were plated on cover slips (R1 and D3 in the presence of feeders, CGR8 in the absence of feeders). Two days after plating, cells were fixed in acetone/methanol, permeabilized with Triton-X-100 and incubated with the anti-p53 antibody Pab 421 or only with blocking buffer, for control. After incubation, cover slips were washed and incubated with an antibody directed against mouse IgG, coupled to Alexa-Fluor-488 (green). To visualize the nuclei, cover slips were incubated with Draq5 (blue). [file 1471-2121-10-46-S1.jpeg]

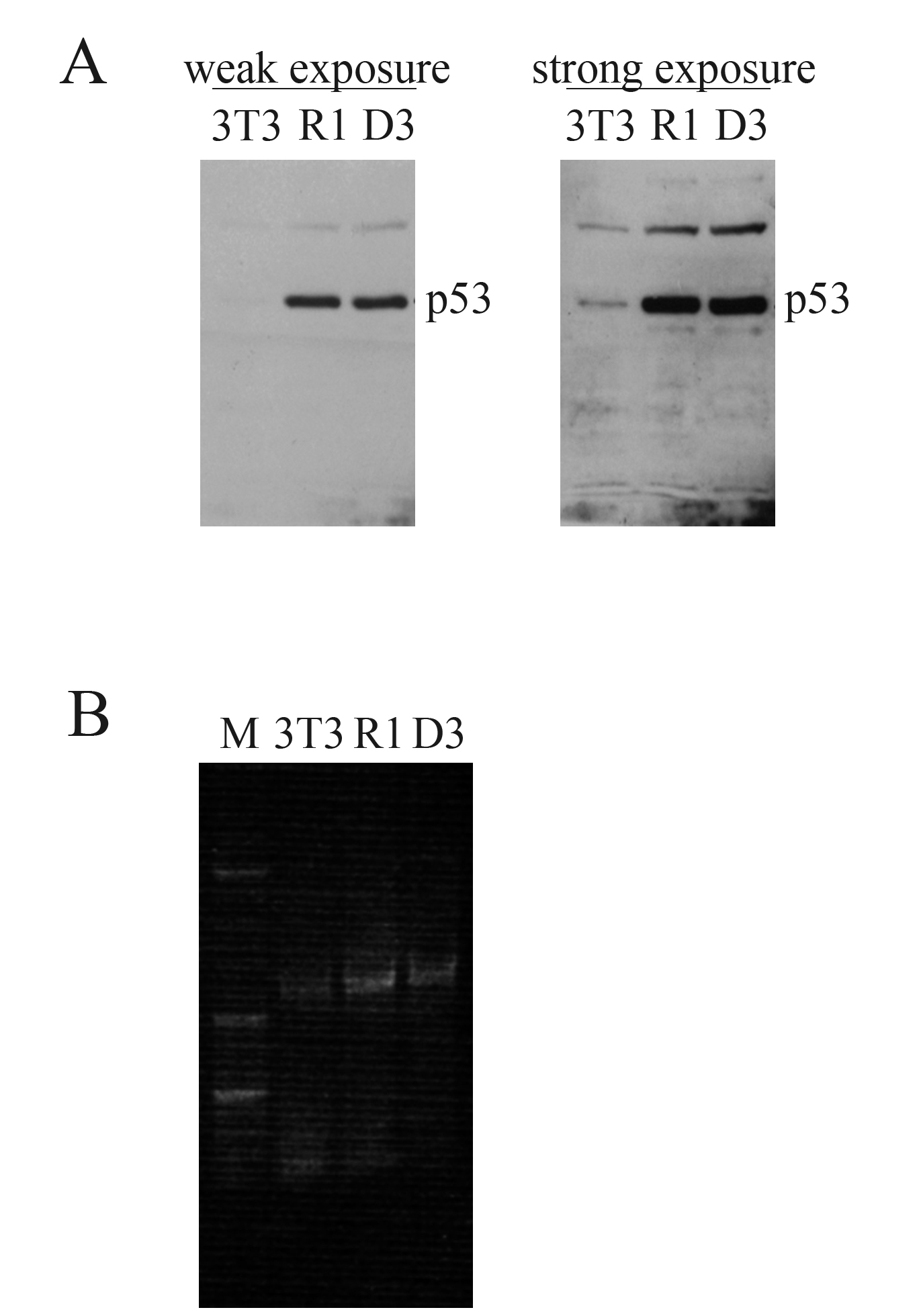

Supplement: Additional file 2 — The 53 kDa-form is the major form of p53 in mouse ES cells. (A) R1, D3 and 3T3 cells, for control, were lysed and 50 μg of the proteins were separated by a 10% SDS-PAGE gel. The proteins were blotted onto an Immobilone P membrane and hybridised with the polyclonal anti-p53 antibody CM5. After washing and hybridisation with an anti-rabbit antibody coupled to HRP, the blot was developed by ECL. (B) Total RNA was prepared from D3, R1 and 3T3 cells, for control, and transcribed into cDNA. PCR was performed using gene specific primers for murine p53 and analysed by agarose gel electrophoresis. [file 1471-2121-10-46-S2.jpeg]

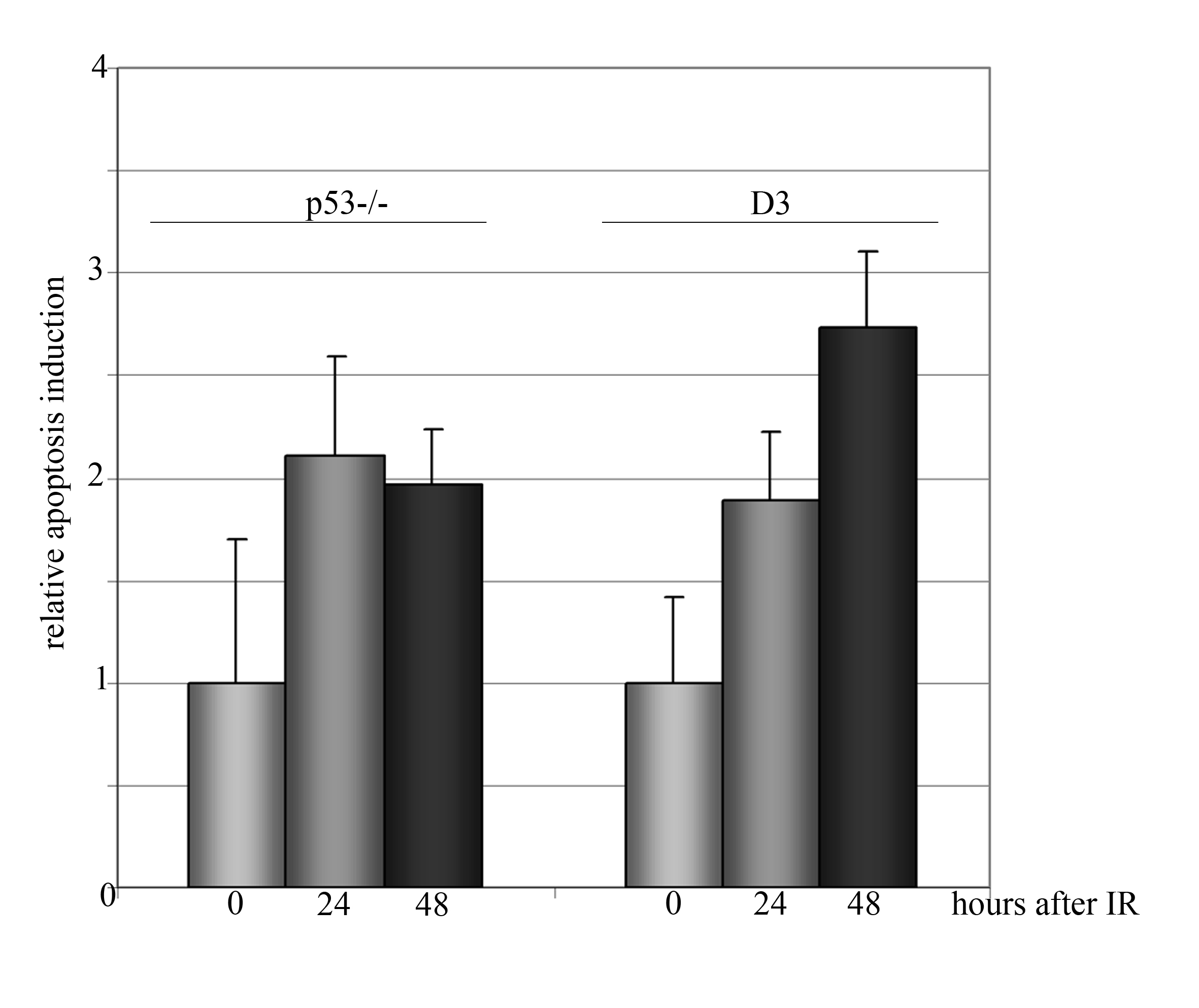

Supplement: Additional file 3 — Annexin-V staining of irradiated p53-/- and p53+/+ ES cells. P53-/- cells and D3 cells that had been passaged twice without feeders (P2 cells) have been irradiated with 2 Gy and harvested after 24 and 48 hours. Cells were resuspended in Ca-containing buffer in the presence of Annexin V-FITC and propidium iodide and analysed by a BD FACScan Flow Cytometer. The data shown in the graph represent the relative number and standard deviation of Annexin-V positive cells of three independent experiments. [file 1471-2121-10-46-S3.jpeg]
